# p63 in skin homeostasis and disease: molecular mechanisms and therapeutic potentials

**DOI:** 10.1038/s41420-026-03060-8

**Published:** 2026-03-24

**Authors:** Yujia Cong, Zhenglin He, Hanming Hao, Haoran Chen, Anqi Chen, Chunyi Li, Yue Hu, Xianling Cong

**Affiliations:** 1 China-Japan Union Hospital of Jilin University, Jilin University, Changchun, China; 2 Department of Dermatology, China-Japan Union Hospital of Jilin University, Changchun, China; 3 College of Basic Medical Sciences, Jilin University, Changchun, China; 4 Institute of Antler Science and Product Technology, Changchun Sci-Tech University, Changchun, China; 5 Department of Biobank, China-Japan Union Hospital of Jilin University, Changchun, China

**Keywords:** Developmental biology, Diseases

## Abstract

As a pivotal member of the p53 family, the p63 gene plays an indispensable role in skin homeostasis and development. The gene encodes multiple isoforms, primarily TAp63 and ΔNp63, which differentially regulate cell proliferation, differentiation, and stress responses through complementary mechanisms. This comprehensive review systematically examines the molecular mechanisms and cellular functions of p63 in cutaneous biology, with particular emphasis on its dual roles in maintaining skin integrity and contributing to disease pathogenesis. We detail the essential functions of p63 in skin development, encompassing epithelial fate determination, chromatin remodeling, cell adhesion, and barrier formation, while also exploring its involvement in skin regeneration and differentiation via metabolic reprogramming and stem cell modulation. Furthermore, we analyze how mutations in p63 underlie ectodermal dysplasia and various dermatological disorders, underscoring the gene’s fundamental importance in cutaneous health. By integrating current knowledge of the diverse biological roles of p63 and its associated therapeutic potential as a target, this review highlights its central significance in both skin physiology and pathology.

## Facts

1. p63 plays a crucial role in skin epidermal development, homeostasis, and regeneration through distinct TAp63 and ΔNp63 isoforms.
2. Isoform-specific functions of p63 establish a dichotomy between proliferative maintenance (ΔNp63) and stress-induced tumor suppression (TAp63).
3. TP63 mutations cause ectodermal dysplasias with distinct clinical phenotypes based on affected protein domains.

## Introduction

The transcription factor p63, a member of the p53 gene family, serves as a master regulator of epidermal development, homeostasis, and repair [1–3]. Classical studies established that p63 deficiency in mice leads to severe defects, including the absence of the epidermis and its appendages (e.g., hair, sweat glands) [4]. In humans, germline mutations in the TP63 gene represent the primary cause of ectodermal dysplasia (ED)-associated disorders, such as Ectrodactyly-Ectodermal dysplasia-Clefting (EEC) syndrome [2, 5]. These disorders are frequently characterized by severe skin abnormalities, craniofacial malformations, and limb defects [2]. Beyond its fundamental role in development, recent research reveals that p63 is pivotal for maintaining epidermal stem cell activity, tissue homeostasis, and driving regeneration and wound repair [6–8]. Notably, p63 is essential for both preserving stem cell identity and promoting cellular differentiation [9, 10]. Understanding how p63 dynamically integrates signaling pathways and orchestrates complex gene regulatory networks is therefore crucial for elucidating normal epidermal biology and its dysregulation in disease.

This review aims to summarize p63’s core functions in skin biology, provide an in-depth analysis of the skin disorders caused by its dysfunction, and evaluate the latest research advances in the field. Deepening our understanding of p63’s complex regulatory networks in both physiological and pathological epidermal processes will advance knowledge of normal skin development, homeostasis maintenance, and the pathological basis of related diseases. Additionally, it will provide a solid theoretical foundation for identifying potential therapeutic targets and guiding future research directions.

## p63 isoforms in skin

**Fig. 1:**
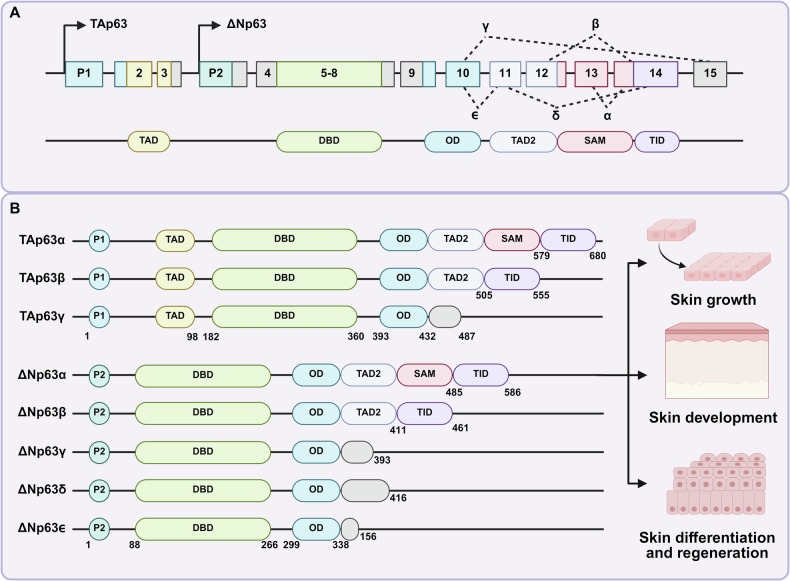
General features and functional roles of p63 isoforms in skin-related processes. **A** Schematic illustration of the alternative promoters generating TAp63 and ΔNp63 isoforms, together with the structural organization and domains of the p63 protein variants. The depicted domains include the transactivation domain (TAD), DNA-binding domain (DBD), oligomerization domain (OD), sterile alpha motif (SAM), and trans-inhibitory domain (TID). **B** Schematic representation of various TAp63 and ΔNp63 isoforms. Among isoforms, the main roles of ∆Np63 in skin processes include skin growth, skin development, and skin differentiation and regeneration.

p63, highly homologous to p53 (TP53) and p73 (TP73), was first discovered in 1997 and identified as a member of the p53 gene family the following year [11]. Subsequent studies found that through the use of alternative promoters and splicing mechanisms, it generates two main classes of isoforms: TAp63, containing the full N-terminal TAD, and ΔNp63, lacking this domain (Fig. 1A) [12, 13]. By the differences in C-terminal splicing, the two subtypes were further divided into three variants: α, β and γ, forming six major subtypes (TAp63α/β/γ, ΔNp63α/β/γ) (Fig. 1B) [14]. Among them, the α subtype contains a complete C-terminal domain (SAM domain), while the C-ends of β and γ subtypes are shorter, which leads to significant functional differences between the subtypes. For example, the α subtype plays a key role in epidermal development and stem cell maintenance, while the γ subtype is more active in DNA damage response [15, 16]. In the 2000s, with the deepening of the study on the function and structure of p63, it was found that ΔNp63 is not only an inhibitor of TAp63, but also can independently regulate epidermal differentiation genes such as KRT14 [17, 18]. The intricate structure of its DNA-binding domain (DBD), which, together with its N-terminal transactivation domain (TAD) and oligomerization domain (OD), constitutes a complex structural system that cooperatively regulates gene expression and cellular activities [12, 19–21]. Several special p63 subtypes have also been identified in recent decades. At the N-terminus of the p63 protein, a substitution of the translation initiation site in the fourth exon produces a ΔΔN subtype that lacks the first 26 amino acids of the ΔN subtype in epidermal keratinocytes [22]. In 2009, Mangiulli et al. first identified ΔNp63δ and ΔNp63ϵ, the first variant derives from the skipping of exons 12 and 13, while the second variant is generated by a premature transcriptional termination in intron 10 [23]. Furthermore, using a start site in exon 1, TA*p63α has an N-terminal extension of 39 amino acids. A similar isoform is GTAp63α, which differs from TA*p63α by replacing the first 21 amino acids with 19 unique residues [24]. Notably, these isoforms are primate-specific and exhibit tissue-restricted expression patterns. The discovery process of p63 subtypes reflects the transformation from “p53 analog” to “multifunctional regulatory factor”, and future studies need to further clarify the precise regulatory mechanisms of each subtype in specific tissues.

Current research widely recognizes ΔNp63, particularly the ΔNp63α isoform, as the predominant isoform in regulating skin-related processes (Fig. 1B). Among them, phenotypes α, β and δ were expressed higher in keratinocytes, while phenotypes γ and ϵ were expressed higher in muscle tissue [6, 7, 23]. Specifically, ΔNp63α maintains the balance between epidermal stem cell proliferation and differentiation by directly regulating key target genes (e.g., KRT14, PERP). Its functional mechanisms include inhibiting cell cycle arrest genes (e.g., p21) to promote basal cell proliferation and maintain the stem cell pool [25, 26], activating glycolysis-related genes (e.g., HK2, PFKFB3) to meet the metabolic demands of highly proliferative cells [27, 28], and acting as a pioneer factor by binding chromatin and recruiting chromatin remodelers (e.g., SATB1, BRG1) to shape the epidermal enhancer landscape and epigenetic state [29]. The overexpression of Np63δ and ϵ isomers, like ΔNp63α, increases the cell proliferation rate, while ΔNp63β, ΔNp63γ and p53 decrease the proliferation rate [23]. In contrast to ΔNp63, TAp63 is typically expressed at low levels in normal epidermis. Under conditions of DNA damage or cellular stress, TAp63 promotes metabolic adaptation by regulating cell cycle checkpoints, antioxidant gene expression, and various metabolic pathways, which contribute to maintaining the quiescent state of skin precursor cells [30, 31].

As the master regulator of epidermal development, p63 regulates a series of critical biological processes through the precise regulation of downstream target gene networks, including cell survival, stem cell self-renewal, migration, differentiation, proliferation, and epithelial-mesenchymal transition [3, 8, 9, 32–34]. Consequently, loss or abnormality of p63 function has severe consequences for epidermal development. For example, Germline mutations in the TP63 gene cause a spectrum of severe ED syndromes. Their characteristic phenotypes often include significant skin defects, accompanied by limb malformations, orofacial clefts, and corneal opacities [2]. Mutations associated with EEC syndrome primarily cluster within the DBD, disrupting p63’s DNA-binding ability and causing thin, dry skin and barrier dysfunction [35]. Osterburg et al. proposed that these DBD mutations can be further subdivided based on their molecular mechanisms, such as those affecting direct DNA contact, zinc finger structure, the H2 region, or the dimer interface [5]. On the other hand, mutations affecting the C-terminal SAM or transactivation inhibitory domain (TID) of the α isoform are associated with Ankyloblepharon-Ectodermal Defects-Clefting (AEC) syndrome and Rapp-Hodgkin syndrome (RHS) [36]. Such mutations can cause abnormal splicing of the p63 protein, this structural instability may further interfere with the interaction between p63 and other proteins, disrupt the binding of p63 to transcription factors such as NF-Y, and lead to abnormal transcriptional regulation of G2/M genes in the cell cycle, leading to severe phenotypes like skin erosions and nail dystrophy [5, 37, 38].

Beyond syndromes caused by germline mutations, p63 dysfunction is closely linked to the pathogenesis of other skin diseases. In inflammatory skin disorders, the pathogenesis of atopic dermatitis (AD) involves functional interactions between p63 and the glucocorticoid receptor in keratinocytes and their interplay at genomic binding sites [39]. In the field of skin cancer, p63 exhibits functional duality in cutaneous squamous cell carcinoma (SCC), overexpression of ΔNp63α drives tumor progression by activating signaling pathways like FGFR2 [40]. Conversely, TAp63 exerts tumor-suppressive functions by inducing apoptosis and cell cycle arrest to inhibit malignant transformation [33, 41, 42]. Given the pivotal roles of p63 outlined above in skin development, homeostasis maintenance, and disease pathogenesis, a deep understanding of its specific physiological functions within epidermal cells is paramount.

## The function of p63 in skin cells

### p63 in skin growth

p63 is essential for epidermal development, as its deletion causes severe epidermal defects. Studies have demonstrated its key roles in promoting keratinocyte stem cell proliferation, maintaining progenitor potential, regulating differentiation fate, and orchestrating epidermal renewal during tissue homeostasis [4, 43].

#### Maintenance of epithelial stem cell properties

p63 functions as a vital regulator of epithelial cell adhesion mechanisms and epithelial morphogenesis, which are essential for sustaining the proliferative potential of stratified epithelial stem cells and facilitating processes related to stem cell renewal [44]. Notably, Senoo et al. (2007) first experimentally demonstrated that p63 knockout mice completely lack embryonic epidermis, though residual cells retain differentiation capacity. Subsequent clonal analysis further confirmed that p63 expression is associated with high-proliferative-potential clones and is essential for maintaining the proliferative capacity of epidermal stem cells [45]. Conversely, in p38α knockout mice, elevated levels of the ΔNp63α protein correlate with expansion of the p63-expressing basal epidermal layer and increased numbers of keratinocytes, underscoring p63’s vital role in preserving epidermal stem cell properties and population dynamics [46]. Except for its fundamental role in sustaining proliferative potential, p63 orchestrates epithelial stem cell maintenance through chromatin-level partnerships. In primary human keratinocytes, ChIP-qPCR analysis has shown that endogenous protein Dnmt3a and p63 co-occupy and functionally cooperate at enhancer regions to regulate epidermal stem cell biological processes [47]. Additionally, p63 expressed in skin precursor cells prevents excessive proliferation through transcriptional activation of Cdkn1c, which encodes p57, thereby preserving stem cell properties and functions [13].

**Fig. 2:**
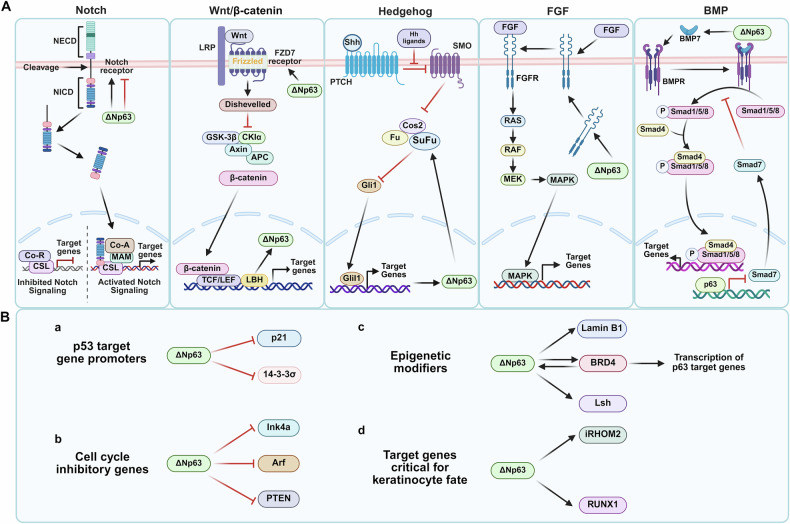
p63-mediated regulation in skin growth. **A** Major developmental signaling pathways intersect with ΔNp63. **B** ΔNp63-regulated molecular programs. **a** ΔNp63 binds to promoters of p53-associated genes to suppress their expression, thereby maintaining cellular proliferation. **b** ΔNp63 represses cell cycle inhibitory genes to regulate cell cycle progression and proliferation. **c** Through interactions with epigenetic modifiers, ΔNp63 influences epigenetic states and target gene transcription. **d** ΔNp63 regulates keratinocyte differentiation and lineage commitment through transcriptional control.

ΔNp63 regulates these programs by context-dependent modulation of developmental signals (Fig. 2A), including Notch [48], Wnt/β-catenin [49], Hedgehog [50], BMP and FGF [51, 52], as well as through regulation of non-coding RNAs such as miR-203 and beta1-adjacent-lncRNA [6, 51, 53]. In epidermal stem cells, ΔNp63 inhibits the Notch signaling pathway to prevent premature differentiation of the cells [54]. Moreover, the involvement of p63 in the Wnt pathway depends on β-catenin, which has both inhibitory and coactivating properties [55]. ΔNp63 facilitates epidermal homeostasis and supports stem cell maintenance by activating the Wnt pathway while inhibiting the differentiation-promoting factor AP1 [56]. Additionally, elevated levels of Axin2, a key marker of the Wnt/β-catenin signaling pathway, were observed in both mouse and human SCC models that had a deficiency in p63/TP63, proving that p63 can inhibit the Wnt/β-catenin signaling pathway in SCC cell lines [57]. Taken collectively, p63 functions as a master coordinator that mediates morphogenesis, cell cycle checkpoints and principal signaling pathways to preserve epithelial stem-cell identity.

#### Maintaining the proliferative potential of basal keratinocytes

In mature epidermis, p63 exhibits a stratified expression pattern, with ΔNp63 predominating in the basal layer [2, 58]. Functioning as a transcriptional repressor, p63 inhibits anti-proliferative target genes to sustain basal epidermal keratinocyte proliferative potential. It is noteworthy that certain p63-mediated cell cycle regulators maintain their expression patterns even in mutated epidermis [53, 59]. Mechanistically, ΔNp63 achieves this pro-proliferative effect through multiple molecular programs (Fig. 2B). Firstly, through promoter interactions, ΔNp63 binds to p53 target gene promoters (including p21 and 14-3-3σ) to suppress their expression, thereby maintaining cellular proliferation [60]. Similarly, p63 represses the expression of cell cycle inhibitory genes and tumor-inhibiting factors (Ink4a, Arf and PTEN). For instance, p63 deficiency leads to upregulated Ink4a/Arf expression and consequent keratinocyte proliferation inhibition [61]. Furthermore, Leonard et al. (2011) showed that ΔNp63α binds to multiple PTEN promoter regions. This interaction promotes Akt activation and cell cycle progression, thereby maintaining cellular proliferative potential [62]. Beyond direct repression, ΔNp63 also engages in cooperative regulation. ΔNp63 nuclear interaction with transcription factor NRF2 at enhancers and promoters of shared target gene CDK12 cooperatively promotes keratinocyte proliferation [8]. Future work should investigate the functional roles of these specific ΔNp63 targets in keratinocyte differentiation within the stratified epithelium. It will also be important to explore strategies modulating key ΔNp63 pathways (e.g., Akt, CDK12) and identify their downstream effectors across epidermal layers for potential therapies targeting proliferation-related skin disorders.

Additionally, p63’s genomic regulation in epidermal keratinocytes involves epigenetic mechanisms extensively. It directly controls the expression of genome organizer protein and ATP-dependent chromatin remodeling genes (Lamin B1, LSH) in keratinocytes [59, 63]. For instance, p63 interacts with BRD4 to synergistically regulate the transcription of p63 target genes (e.g., HK2, FOXM1 and EVPL) in keratinocytes. When p63 was knocked out, the transcription levels and protein levels of those genes were significantly reduced [64]. The loss of p63 also leads to changes in enhancer methylation levels in prostate basal epithelial cells, which affect gene expression [65]. Beyond these epigenetic modifications and chromatin remodeling, ΔNp63 also exerts direct transcriptional control over specific target genes critical for keratinocyte fate [2]. Specifically, overexpression of the p63 isoforms significantly induced iRHOM2 expression at mRNA and protein levels. ΔNp63α, the major p63 isoform expressed in epidermis, likewise serves as the principal regulator of iRHOM2 in keratinocytes [66]. Moreover, ΔNp63 can bind RUNX1 intron 5 and exon 1 DNA sequences. This binding enables ΔNp63 to positively regulate RUNX1 expression in proliferating human keratinocytes, while negatively controlling it in cells that have exited the cell cycle and initiated differentiation [67]. By regulating glycolysis metabolism, ΔNp63 maintains the energy metabolism and proliferative potential of basal keratinocytes. Experimental results demonstrate that ΔNp63 knockdown significantly reduces the expression of glycolysis regulatory enzyme PFKFB3, leading to weakened glycolytic metabolism [28]. These studies provide evidence of ΔNp63 control of keratinocyte proliferation, migration and differentiation. Critically, p63 plays a pivotal role in sustaining the proliferative potential of basal keratinocytes through multifaceted transcriptional repression of key anti-proliferative genes and cell cycle inhibitors, alongside its involvement in epigenetic regulation and activation of pro-proliferative pathways (Fig. 2B).

### p63 in skin development

**Fig. 3:**
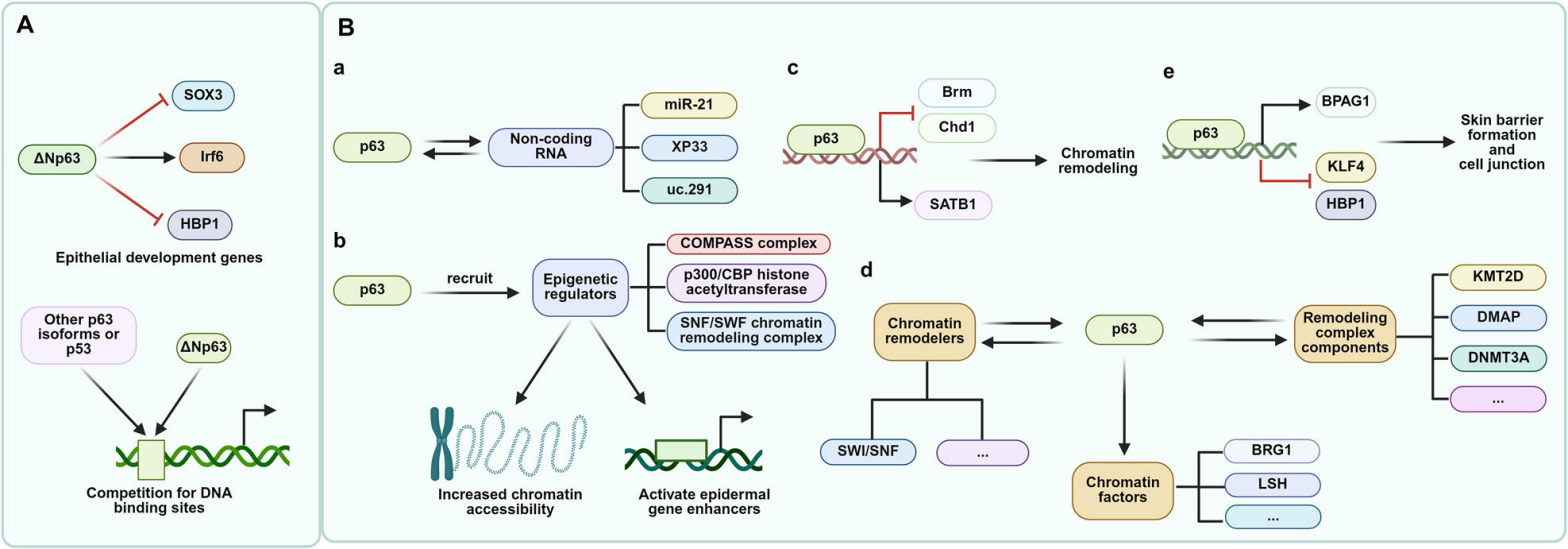
The role of p63 in regulating epithelial development and epidermal gene expression. **A** ΔNp63 directly regulates epithelial development genes, while other p63 isoforms or p53 compete with ΔNp63 for these binding sites, influencing the expression of target genes crucial for epithelial development. **B a** p63 regulates non-coding RNAs, which in turn impact gene expression during epidermal development. **b** p63 recruits epigenetic regulators to increase chromatin accessibility and activate epidermal gene enhancers. **c** p63 interacts with factors to drive chromatin remodeling. **d** p63 shapes the chromatin landscape during epidermal development via interactions with chromatin remodelers. p63 also promotes various chromatin factors. Moreover, p63 interacts with remodeling complex components to promote chromatin processes. **e** p63 controls genes involved in skin barrier formation and cell junctions.

During cutaneous development, p63 functions from embryogenesis onward to orchestrate the differentiation of monolayer embryonic epidermis into mature, stratified epidermis. Through gene regulation and other mechanisms, p63 guides the differentiation of epidermal cells into functionally distinct layers and epidermal appendages (Fig. 3).

#### Epithelial cell development

p63 regulates cutaneous development through modulation of epithelial development genes while maintaining normal tissue homeostasis [68]. During early embryogenesis, p63 safeguards epithelial lineage commitment by preventing Sox3 binding to neural gene enhancers, simultaneously activating epidermal genes and repressing non-epithelial genes [69]. It is generally agreed that p63 forms a complex regulatory network that coordinates epidermal development and homeostasis by regulating genes such as Irf6 and HBP1. For instance, p63 deficiency leads to a significant downregulation of Irf6 expression, which plays a key role in epidermal differentiation and intercellular adhesion [70]. Additionally, p63 promotes the proliferation of the epidermal basal layer by directly inhibiting the expression of HBP1 [44]. Beyond repression, p63 was shown to maintain the FGF signaling pathway, thereby retaining the proliferation capacity of epithelial stem cells [71]. Ferone et al. illustrated that the p63 mutation resulted in reduced expression of FGFR2 and FGFR3, which in turn affected FGF signaling and led to epidermal stem cell proliferation defects [53].

In addition to acting directly on epithelial development-related genes, ΔNp63 regulates epithelial development through competition with other p63 isoforms or p53 for DNA-binding sites. For instance, both ΔNp63 and TAp63 can bind to the same DNA sequences. ΔNp63 often acts as a transcriptional repressor, counteracting the strong transactivation potential of TAp63. This competitive interaction ensures a balanced regulation of epithelial gene expression, which is critical for normal development and homeostasis [72]. Moreover, wild-type ΔNp63α strongly inhibits p53 reporter gene transactivation, thus playing a crucial role in epithelial development gene regulation [73]. Thus, it can be proposed that p63 orchestrates epidermal development and homeostasis through a multifaceted regulatory network.

#### Chromatin remodeling during epidermal development

p63 influences epidermal development through non-coding RNA regulation (e.g., miR-21, XP33, uc.291) [74–76]. Functioning as an epidermal pioneer factor that opens chromatin regions, p63 binds compact chromatin and recruits epigenetic regulators (COMPASS complex, p300/CBP histone acetyltransferase, SNF/SWF chromatin remodeling complex) to increase chromatin accessibility and activate epidermal gene enhancers [10, 69, 77–80]. p63 knockdown alters expression of numerous chromatin remodeling genes (e.g., Satb1 downregulation; Chd1 and Brm upregulation) [69], which proves that p63 can influence epidermal development by regulating chromatin remodeling. During epidermal development, p63 collaborates with chromatin remodelers (e.g., mammalian SWI/SNF) to control the chromatin landscape [78, 81, 82]. p63 directly regulates various chromatin factors, including SMARCA4 (BRG1) and HELLS (LSH) [83, 84], and interacts with multiple remodeling complex components (KMT2D, DMAP, DNMT3A, DNA-dependent ATPases, and DNA decoupling enzymes) to regulate chromatin processes during epidermal development [33, 47, 85]. Taken collectively, p63 orchestrates epidermal chromatin remodeling through pioneer factor activity and collaborative recruitment of epigenetic regulators.

#### Formation of cell layers with different functions

During epidermal development, TAp63 isoforms are expressed first and initiate commitment to epithelial stratification [86–88]. Subsequently, ΔNp63α becomes the predominant isoform; its expression is highest in the basal layer and declines as cells move outward, a gradient that both supports early morphogenetic programs and later permits terminal differentiation while preserving basement-membrane integrity [86–88]. Throughout the stratification process, regulated ΔNp63 expression governs cell differentiation fate, enabling the normal formation and function of all epidermal layers [89]. Thus, p63, predominantly through ΔNp63α, governs epidermal stratification by dynamically balancing progenitor proliferation and layer-specific differentiation.

This regulatory role is underpinned by p63’s control of specific gene networks. Gene expression profiling and chromatin immunoprecipitation analyses have identified a set of p63-regulated genes involved in epidermal stratification. For example, p63 negatively regulates HBP1 expression, thereby indirectly affecting skin stratification [44]. In this respect, Shalom-Feuerstein et al. (2011) demonstrated that only wild-type stratified epidermal cells express proliferation marker Ki67, while p63 knockout cells show no proliferation, proving p63-dependence for embryonic stratification proliferation [90]. Collier et al.’s study in Nature (2022) proposed that during skin development, p63 can cooperate with other transcription factors (such as TFAP2A/C, GRHL2, etc.) to reshape the chromatin landscape through epigenetic modification, thereby inducing its own expression and starting the stratification program of epithelial cells [91]. Collectively, p63 acts as the master regulator coordinating the complex molecular and cellular events, which are essential for generating the functionally distinct layers of the epidermis.

#### Skin barrier formation and skin appendage development

p63 is essential for skin barrier formation and the development of epithelial appendages, such as hair follicles and exocrine glands [59]. During barrier formation, p63 facilitates the maintenance of skin cell junctions and preserves normal structural and functional integrity by upregulating the expression of the junctional gene BPAG1 [92]. In its capacity to promote growth in normal keratinocytes, p63 directly represses KLF4 and HBP1 by binding their promoters [44, 93]. Candi et al. (2006) pioneeringly identified that p63 reintroduction in knockout mice caused varying degrees of epidermal restoration, as evidenced by re-epithelialization, increased expression of markers in the basal and suprabasal layers, and the reappearance of basement-membrane and hemidesmosome structures [89]. Subsequent studies have shown that p63α/β, rather than p63γ, is essential for normal development of skin and limbs [14]. Notably, Nagano et al. (2024) utilized CRISPR-Cas9 technology to create a p63 knockout chimeric mouse, which similarly showed loss of epidermal structure, confirming that p63-deficient keratinocytes fail to form proper skin grafts with functional dermal appendages [94]. These studies have unraveled how p63 governs skin barrier integrity and appendage morphogenesis through junctional regulation and isoform-specific essentiality.

#### Cell adhesion regulation in skin

p63 is a master regulator of gene expression in the epidermis and in other stratified epithelia, positively controlling numerous tissue-specific genes, including those encoding cell adhesion molecules [6, 95, 96]. Through interaction with promoters and enhancers of adhesion-related genes, p63 transcriptionally controls the expression of focal adhesion components [97]. Specifically, ΔNp63α upregulates β4 integrin expression to modulate keratinocyte adhesion and detachment from the underlying mesenchyme. Both TAp63γ and ΔNp63α isoforms maintain epidermal stem cell regenerative capacity by upregulating integrin subunit ITGA3, thereby preventing anoikis [98, 99]. In corneal epithelial progenitor cells, p63 regulates BCAM expression, which is functionally required for cell migration and differentiation [100]. During epidermal development, p63 forms a regulatory network with IRF6 and RIPK4 that coordinates cell–cell adhesion and differentiation. This multistep pathway controls adherens junction and desmosome assembly, where RIPK4 phosphorylates and activates IRF6, subsequently modulating p63 activity to balance proliferation and differentiation [101]. The reciprocal regulation between the adhesion program and p63 function in epidermal maintenance and survival remains an active area of investigation [102]. Mutations in TP63 underlying AEC syndrome cause coordinated downregulation of focal adhesion proteins, impairing keratinocyte adhesion to the basement membrane [97, 103]. Genetic deletion of p63 exon 13 in mice has demonstrated that the C-terminal domain is intrinsically required for epithelial adhesion, as ΔNp63αΔ13/Δ13 mutants exhibit severe skin barrier defects and impaired expression of integrins and desmosomal proteins [104]. The p63L514F SAM domain mutation further disrupts keratinocyte proliferation through oxidative stress and cell cycle alterations, revealing distinct mechanisms by which p63 maintains epidermal integrity [105]. Collectively, p63 serves as a central transcriptional coordinator of cutaneous cell adhesion, directly regulating integrins, hemidesmosomal components, and junctional proteins to maintain epidermal integrity and regenerative capacity.

### p63 in skin differentiation and regeneration

Skin differentiation and regeneration represent critical physiological processes maintaining cutaneous integrity and function. These processes preserve skin barrier function against dehydration, mechanical trauma, and microbial invasion [106]. p63 serves as a central regulator of these processes, with essential roles in both physiological and pathological contexts. This section examines the influence of p63 on skin differentiation and regeneration through metabolic reprogramming, stem cell modulation, and gene expression regulation.

#### Metabolic reprogramming

Metabolic reprogramming enables cellular adaptation to environmental changes by modifying metabolic pathways to meet energy and biosynthetic demands—a key mechanism underlying cellular morphological and functional remodeling [107]. Environmentally, high-glucose conditions promote the expression of p63, while the loss of p63 increases the expression of monounsaturated fatty acid synthesis rate-limiting enzymes, resulting in changes in lipid homeostasis [108].

**Fig. 4:**
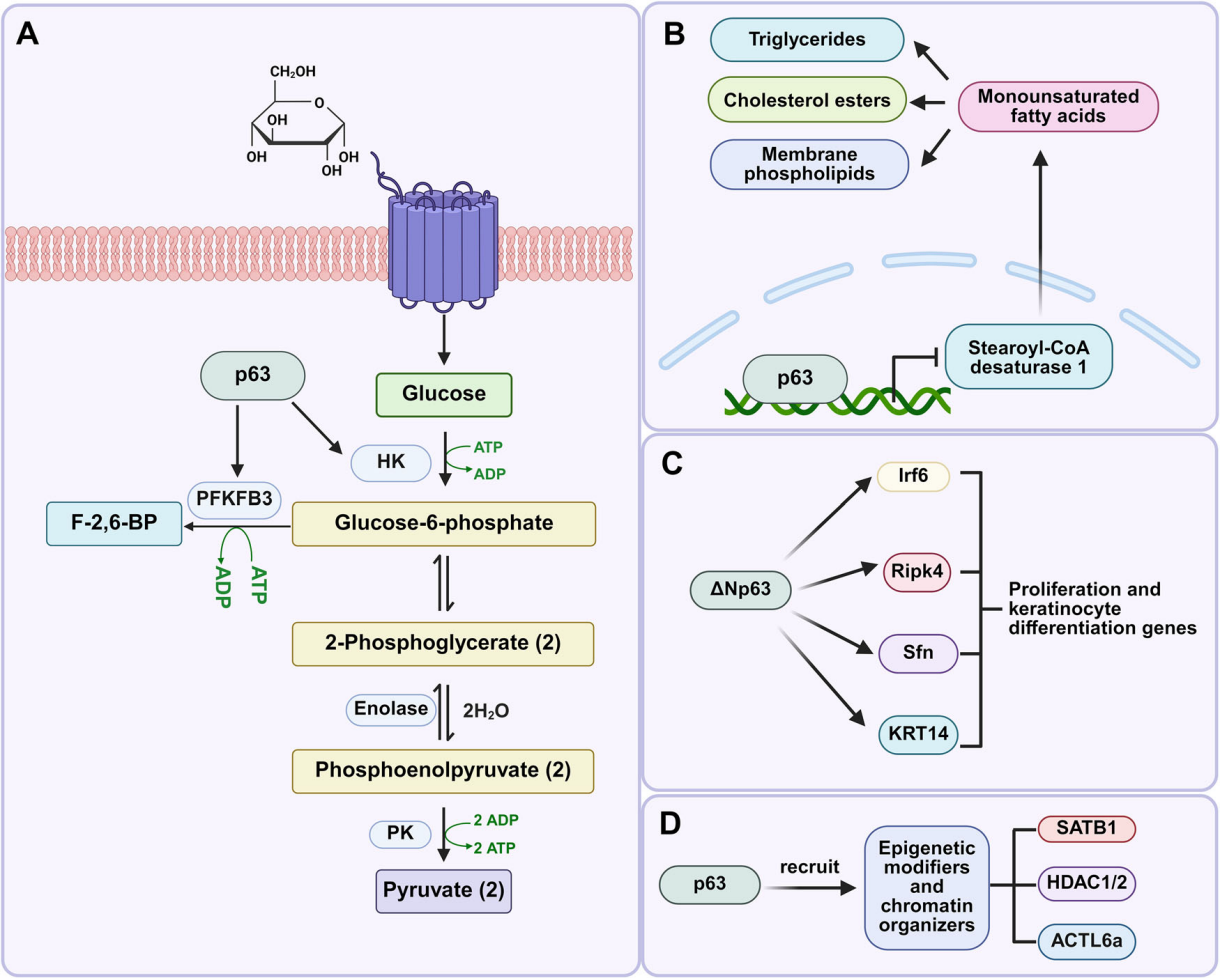
The influence of p63 on skin differentiation and regeneration through metabolic reprogramming and gene reprogramming. **A** p63-mediated regulation of glycolysis. **B** p63-driven monounsaturated fatty acid biosynthesis. **C** ΔNp63 controls target genes associated with proliferation and keratinocyte differentiation through direct transactivation. **D** p63 recruits epigenetic regulators and chromatin remodeling factors to regulate its numerous target genes.

Glucose oxidation represents a metabolic hub, with the modulation of glycolysis effectively controlling differentiation. Hexokinase 2 (HK2) and 6-phosphofructo-2-kinase/ fructose-2,6-bisphosphatase 3 (PFKFB3) are critical regulators of glycolysis. Based on this, p63 emerges as a key metabolic regulator, exerting direct control over HK2 expression, which in turn influences glycolytic flux and energy homeostasis [109]. p63 promotes HK2 expression, reducing oxidative stress and affecting keratinocyte polarization. Conversely, a reduction in p63/HK2 activity leads to decreased antioxidant enzyme expression (GPX2, SOD2, NQO1), which impairs mitochondrial respiration, elevates reactive oxygen species levels, and causes mitochondrial membrane hyperpolarization [110, 111]. Viticchiè et al. (2015) demonstrated that p63 ensures the stable expression of HK2 in epidermal cells, enhancing glucose metabolism-oxidative phosphorylation coupling to provide energy for proliferation and protection against oxidative stress [112]. Collectively, p63 acts as a central metabolic regulator in epidermal keratinocytes by controlling the expression of glycolysis regulators (Fig. 4A).

In addition to its role in glycolysis, a recent study (2025) showed that p63α and p63γ can bind to the promoter of stearoyl-CoA desaturase 1, thereby inhibiting its expression. This subsequently suppresses the production of monounsaturated fatty acids, which are key components of triglycerides, cholesterol esters, and membrane phospholipids (Fig. 4B) [27]. Intriguingly, Piro et al. showed that p63-deficient and senescent keratinocytes share metabolic profiles characterized by oxidative stress and changes in lipid metabolism, suggesting p63’s inhibitory role in keratinocyte senescence and lipid metabolism, which unveiled the important metabolic links between p63 and keratinocyte senescence [113]. These findings solidify p63’s role as a key metabolic regulator influencing lipid homeostasis and cellular aging pathways. p63’s metabolic governance safeguards epidermal integrity by preventing lipid dysregulation and keratinocyte senescence. Further studies are needed to establish the link between p63 and cellular metabolism and aging in epidermal cells. Through these metabolic pathways, p63 ensures that skin stem cells and keratinocytes maintain adequate energy and redox balance to support tissue repair and regeneration. p63-driven metabolic reprogramming is thus essential for preserving normal epidermal structure/function and facilitating cutaneous repair.

#### Effects on skin stem cells

Skin stem cells, which are capable of self-renewal and multilineage differentiation, play a vital role in preserving the normal structure and function of the epidermis, facilitating skin repair and regeneration, and maintaining microenvironmental homeostasis [102]. While p63, expressed in various organ stem cells including skin, serves as a critical regulator of epidermal stem cell proliferation and differentiation [114].

EPSC-derived clones show prominent ΔNp63α expression, that are essential to maintain their proliferative and regenerative capacity [115]. Interfollicular stem cells, known as epidermal regenerators, demonstrate elevated levels of p63 expression during the wound healing process, which is crucial for effective healing and regeneration [116]. Furthermore, p63 phosphorylation increases during wound healing, enhancing stability by reducing susceptibility to degradation, which is essential for maintaining continuous proliferation and promoting repair [117].

The microenvironment of stem cells significantly influences the expression and function of p63. In this respect, Wnt, Notch, and BMP pathways all impact p63 expression. Wnt activation promotes skin stem cell proliferation and maintains high p63 (particularly ΔNp63) expression [118], while Notch and BMP inhibit p63 expression to promote differentiation [119]. Additionally, the ubiquitin-proteasome system negatively regulates p63 isoforms through proteasomal degradation and ubiquitination, affecting proliferation and differentiation [120]. Collectively, these mechanisms highlight the essential role of p63 as a central regulator of skin stem cell self-renewal and regenerative capacity.

#### Gene reprogramming and regulation of gene expression

Regulation of gene expression is fundamental to cellular differentiation and regeneration. Genetic reprogramming offers a window into cellular plasticity, an important facet of research in regenerative medicine. Within this context, p63 acts as a master regulator of epithelial genes, sustaining basal cell proliferation while preventing premature differentiation during wound repair and lineage commitment [2]. It controls target genes through direct transactivation or protein-protein interactions. Several proliferation and keratinocyte differentiation genes (Irf6, Ripk4, Sfn, KRT14) are identified as direct p63 targets (Fig. 4C) [121–123]. Recent studies also indicate the interaction between p63 and bromodomain-containing protein 4, an established epigenetic modulator and transcriptional coactivator [64]. Furthermore, ΔNp63α protein activates epidermal stem cell self-renewal by specifically binding to the p63-responsive element in the CCND1 gene (encoding cyclin D1). Critically for tissue regeneration, p63 also interacts with TEAD family proteins to form functional complexes that bind to YAP target genes’ regulatory regions, significantly enhancing their transcriptional activity, which is crucial for skin regeneration [124, 125]. Collectively, these direct transcriptional interactions and partnerships with co-regulators like BRD4 and TEAD/YAP establish p63 as a master coordinator of the gene expression networks essential for keratinocyte proliferation, differentiation, and ultimately, skin regeneration.

Beyond direct transcriptional control, p63 orchestrates a comprehensive interactome network comprising transcriptional co-activators, chromatin remodelers, and signaling adapters to regulate epidermal gene expression programs [126]. For instance, nuclear matrix protein SATB1, a p63 downstream target, contributes to the establishment of region-specific epigenomic modifications that are critical for the regulation of epithelial adhesion genes (ITGA3, lamininγ2, P-cadherin) [127]. Additionally, Gatti et al. (2022) demonstrated that ΔNp63 functions as a transcriptional repressor by recruiting histone deacetylases HDAC1 and HDAC2, or interacting with epigenetic modification factor ACTL6a [128]. Thus, through the recruitment of diverse epigenetic modifiers and chromatin organizers like SATB1, HDAC1/2, and ACTL6a, p63 orchestrates crucial chromatin-level changes that govern the complex transcriptional programs underpinning epidermal differentiation and barrier function (Fig. 4D). In summary, p63 serves as a central regulator of skin differentiation and regeneration through regulation of metabolic pathways, modulation of stem cell behavior, and control of gene expression (Table 1**)**. Its multifaceted roles underscore the importance of p63 in skin homeostasis and suggest possible therapeutic targets for cutaneous disorders.

**Table 1:** Summary of p63 ChIP-seq and ATAC-seq experiments in skin homeostasis and disease.

Gene	Organism	Mechanism	Sequencing technique	GSE accession	Reference
ΔNp63β	Homo sapiens	ΔNp63β regulates both canonical ΔNp63ɑ targets and a unique set of genes with varying biological functions.	ChIP-seq	GSM8660764	[159]
ΔNp63	Homo sapiens	Chromatin modification is not a prerequisite for p63 binding, but p63 binding can elicit chromatin remodeling.	ChIP-seq	GSE140329	[29]
TAp63 and ΔNp63	Mus musculus	TAp63 and ΔNp63 physically and functionally interact with distinct transcription factors for the downstream regulation of their target genes, thus ultimately leading to the regulation of unique transcriptional programs and biological processes.	ChIP-seq	GSM4443817 GSM4443818	[160]
p63	Homo sapiens	p63 is a key mediator of epidermal development. Enhancers established by p63 are highly enriched for single-nucleotide polymorphisms associated with non-syndromic cleft lip ± cleft palate (CL/P).	ChIP-seq /ATAC-seq	GSM3597654 GSM3597655 GSM3597676 GSM3597677	[82]
p63	Homo sapiens	p63 effects major transcriptional changes only after morphogens alter chromatin accessibility, establishing an epigenetic landscape for p63 to modify.	ATAC-seq	GSM3455870	[81]
p63	Homo sapiens	Cooperation of p63 and CTCF seemed to assist chromatin interactions between p63-bound enhancers and gene promoters in skin keratinocytes.	ATAC-seq	GSM3509946	[77]
p63	Homo sapiens	Mutant p63 rewires the enhancer landscape and affects epidermal cell identity, consolidating the pivotal role of p63 in controlling the enhancer landscape of epidermal keratinocytes.	ChIP-seq	GSM2597280 GSM2597281 GSM2597282	[161]
p63	Homo sapiens	The role of VDR involves cross-talk with the epidermal master regulator p63 through super-enhancer-mediated epigenetic dynamics.	ChIP-seq	GSM7226965 GSM7226967	[162]

### p63-associated skin disorders

**Fig. 5:**
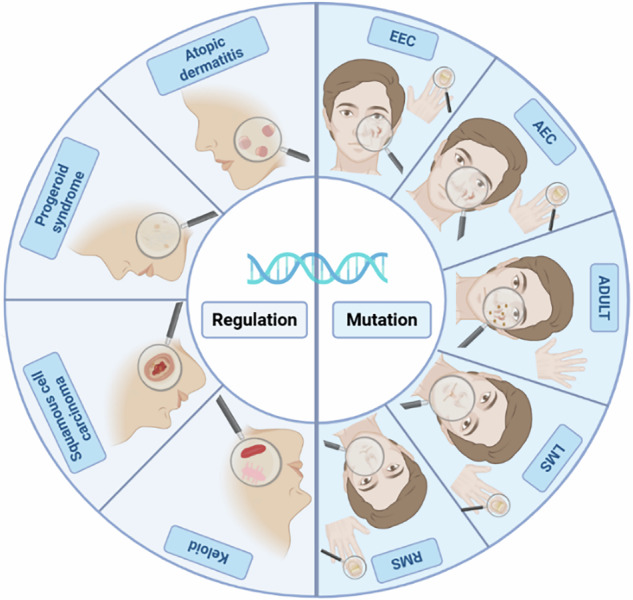
Cutaneous manifestations of diseases related to p63 gene. Mutations in the p63 gene and dysregulation of its expression represent core mechanisms driving the pathogenesis of diverse dermatological conditions.

Mutations in the p63 gene and dysregulation of its expression represent core mechanisms driving the pathogenesis of diverse dermatological conditions (Fig. 5). These genetic alterations can induce loss-of-function or gain-of-function modifications that disrupt ectodermal developmental programs, leading to hereditary disorders exemplified by EEC syndrome [129]. At the regulatory level, imbalanced expression of TAp63 and ΔNp63 isoforms critically influences keratinocyte fate determination, thereby contributing to the pathogenesis of multiple skin diseases [130]. Comprehensive delineation of p63’s mutational landscape and regulatory networks is fundamental for elucidating pathological mechanisms, advancing precision diagnostics, and identifying novel therapeutic targets.

### p63 mutations in ectodermal dysplasia

p63 mutations underlie clinically heterogeneous genetic disorders [131]. Approximately 80% of mutations localize to functionally critical domains—specifically the DBD and OD—causing ED syndromes, a spectrum of conditions characterized by aberrant ectodermal development [114]. This disease group includes EEC syndrome, AEC syndrome, Acro-Dermo-Ungual-Lacrimal-Tooth (ADULT) syndrome, limb mammary syndrome (LMS), and RHS [132], all demonstrating significant genotype-phenotype correlations (Table 2).

**Table 2:** Clinical characteristics and mechanisms of ED.

Diseases	Clinical characteristics		Mechanisms	Reference
	Skin	Related manifestations		
EEC syndrome	Xerotic fragile skin, sparse coiled hair, and onychodystrophy	Limb malformations	DBD mutations	[133]
AEC syndrome	Xerotic fragile skin, sparse coiled hair, and onychodystrophy	Eyelid fusion	SAM domain mutations	[129, 133]
ADULT syndrome	Generalized freckling, dry and atrophic skin, onychodystrophy	Ectrodactyly and syndactyly, lacrimal duct anomalies, hypodontia	R298 mutation 243W mutation	[163, 164]
LMS	Palate facial clefting, nail dysplasia	Ectrodactyly with bilateral mammary hypoplasia and hypoplastic nipples, hypodontia	R204Q mutation R227Q mutation	[33]
RMS	Coarse and curly hair, nail dysplasia	Cleft lip and palate, small mouth, narrow nose.	1709DelA mutation R279H mutation	[165]
Mosaic expression	Hypopigmented Blaschkoid macules with alopecia	Dental anomalies	Copy number variations	[142]

*EEC* ectrodactyly-ectodermal dysplasia-cleft lip/palate, *AEC* ankyloblepharon-ectodermal defects-cleft lip/palate, *DBD* DNA-binding domain, *SAM* sterile alpha motif, *ADULT* Acro-Dermato-Ungual-Lacrimal-Tooth, *LMS* limb mammary syndrome, *RHS* Rapp-Hodgkin syndrome.

EEC and AEC syndromes share autosomal dominant inheritance and common phenotypic features such as xerotic fragile skin, sparse curly hair, and nail dystrophy [133], yet exhibit distinct clinical profiles. EEC syndrome is defined by severe limb malformations [134], with DBD hotspot mutations like R204, R227, R279, R280, and R304, demonstrating functional consequences, which targeted ablation of the ΔNp63-R304W allele restores wild-type transcriptional activity, delaying cutaneous erosions and corneal dysfunction in patients [135]. Conversely, AEC syndrome primarily manifests with ankyloblepharon, where mutant proteins undergo pathological aggregation, causing functional loss [136]. The pivotal role of p63 in craniofacial morphogenesis is further substantiated by two key observations: in non-syndromic cleft lip/palate (nsCL/P), p63-bound enhancers densely populate craniofacial development-associated SNP regions [82], while ΔNp63α mutations cause limbal stem cell deficiency resulting in corneal epithelial degeneration and vision loss [137].

ADULT syndrome patients typically carry R298Q or Q243W mutations, presenting ectrodactyly/syndactyly combined with classic ectodermal defects, including lacrimal duct anomalies, hypodontia, and nail dystrophy, which were often accompanied by generalized freckling and cutaneous atrophy [138]. LMS, predominantly associated with R204Q or R227Q mutations, features ectrodactyly with bilateral mammary hypoplasia and nipple aplasia [139], exhibiting minimal skin alterations and palatal-restricted facial clefts when present [140]. It may also be accompanied by nail dysplasia, and hypodontia [141]. RHS is a rare ED variant linked to 1709DelA or R279H mutations, which demonstrates variable manifestations: cleft lip/palate, microstomia, narrow nasal configuration, frequently with wiry curly hair with increasing alopecia risk over time and nail dysplasia [140]. Notably, a distinct ED subtype manifests as hypopigmented Blaschkoid linear macules with alopecia and dental anomalies, its phenotypic variability explained by mosaic p63 copy-number variations [142]. Collectively, these findings establish p63 as a master regulator of ectodermal development, whose functional disruption reveals profound complexity through diverse clinical phenotypes.

### p63 regulation in skin diseases

Dysregulated p63 signaling plays a central role in acquired dermatological conditions. In mature epidermis, p63 dysfunction compromises barrier integrity through epidermal disintegration and chronic inflammation, driving AD pathogenesis [129]. Clinically, this often manifests as erythema, edema or papular eruptions, along with excoriation and lichenification [143]. Progeroid syndromes, a rare genetic disorder mimicking accelerated aging, result from coordinated dysregulation of DNA replication timing and p63 expression [144], impairing epithelial differentiation and homeostasis to cause adolescent-onset osteopenia, scleroderma-like changes, and lipodystrophy [145].

Cutaneous squamous cell carcinoma (cSCC), a common non-melanoma skin malignancy, typically presents as a nodular, red lesion, associated with ulceration or crusting of the epidermis [146]. cSCC reveals the stage-dependent duality of p63 function, while TP63 ablation in advanced tumors induces regression, confirming its tumor maintenance role [40], deletion during early carcinogenesis accelerates progression [45], unmasking a context-dependent tumor-suppressive function [147]. This paradox necessitates stage-specific therapeutic strategies for targeting p63 in cSCC.

It is implicated that FGFR2 alternative splicing coupled with p63 downregulation in keloid pathogenesis, mediating aberrant epidermal remodeling and adnexal regeneration failure [148]. Given p63’s pleiotropic roles in skin biology, elucidating disease-specific regulatory mechanisms and developing pathway-directed therapies remain critical research priorities. Comprehensive phenotypic and molecular profiles of p63-associated disorders are systematically summarized in Table 3.

**Table 3:** Skin diseases associated with p63.

Diseases	Phenotypes	Clinical characteristics and related features	Mechanisms	Reference
AD	Extrinsic AD	Recurrent, erythematous skin lesions, intensely pruritic	IL-33 signaling activation and Th1, Th2 polarization	[145, 166]
	Intrinsic AD			
Progeroid syndrome	HGPS	Alopecia, loss of body fat, limited growth, scleroderma, and cardiovascular complications	Abnormal DNA replication timing, LMNA mutations	[167, 168]
Cutaneous SCC	Well-differentiated SCC: keratoacanthoma, verrucous carcinoma.	Risks include light skin, age, male gender, exposure to sunlight, immunosuppression, HPV, chronic scarring conditions, familial cancer syndromes, and environmental exposures	TP53, CDKN2A, Ras, and NOTCH1 mutations	[40, 169]
	Poorly-Differentiated SCC: desmoplastic cutaneous squamous cell carcinoma, adenosquamous cutaneous squamous cell carcinoma, and cutaneous squamous cell carcinoma associated with scarring processes			
Keloid	—	Over-proliferation of fibroblasts, an increase of collagen production.	ΔNp63 abnormal expression, TGF-β1 and TGF-β2 overexpression	[131, 170]
		Firm, mildly tender, bosselated tumors with shiny surface and telangiectasia		

*AD* atopic dermatitis, *HGPS* Hutchinson–Gilford progeria syndrome, *SCC* squamous cell carcinoma, HPV human papillomavirus, *CDKN2A* cyclin-dependent kinase inhibitor 2A, *TGF-β* transforming growth factor-beta.

### Potential therapeutic implications

The pleiotropic and genetically heterogeneous nature of p63-related syndromes has long posed a major challenge to effective treatment. Beyond surgical correction and supportive care, no curative therapy currently exists for disorders caused by TP63 mutations [2, 33]. However, recent advances are opening fresh perspectives for improving the management of these conditions.

Pharmacological strategies are exploring small-molecule compounds that potentiate the activity of the residual wild-type p63. APR-246/PRIMA-1^MET^ was the first compound reported to partially restore the function of the molecular pathways downstream of p63 that are disrupted in EEC patients. Yet its efficacy is allele-restricted and systemic exposure limits chronic use [149–151]. Moreover, the severe cutaneous phenotype in patients with AEC syndrome may be caused by the binding of AEC mutants to p63. Restoring the protein clustering function in AEC syndrome may pave a new way for future treatments [136]. Missense alleles that destabilize the DNA-binding or SAM domain dominate the mutational landscape. Recently, Boncimino et al. (2025) showed that low-dose anthracyclines (doxorubicin and diMe-doxorubicin) disaggregate misfolded p63-SAM mutants, restore monomeric p63 and rescue epidermal differentiation in both AEC-patient keratinocytes and a ΔNp63α-L514F mouse model [152]. In this regard, a topical analog (diMe-doxorubicin) retains activity without the cardiotoxic scaffold, suggesting that anthracyclines may serve as a valuable starting point for developing a therapy aimed at rescuing p63 function by preventing aggregation-prone AEC-linked p63 mutants.

Skin diseases with their origin in p63 mutations exhibit marked clinical or genetic heterogeneity, necessitating personalized approaches. For example, EEC syndrome frequently involves limbal stem cell deficiency (LSCD)-induced corneal defects [153]. A study through stem cell therapy showed that heterozygous R311K-p63 stem cells are useful in treatment of such corneal defects [154], highlighting the potential for personalized stem cell therapy for genetic diseases. For the frequent dominant-negative R304W allele, allele-specific siRNA or AAV-delivered CRISPR base-editing normalized transcriptional programs and stratification in patient iPSC-derived skin in vivo [135, 155], demonstrating the therapeutic promise for gene silencing technology in the treatment of genetic disorders.

Recent non-pharmacological photobiomodulation (PBM) using 808 nm near-infrared light upregulates p63 expression, and promotes re-epithelialization, extracellular matrix deposition, and wound healing [156]. Although PBM does not correct the mutant genotype, it reduces inflammatory cytokine expression [157], and enhances barrier integrity by upregulating tight-junction proteins [158]. While formal evidence in p63-related skin disorders is still lacking, these mechanisms support its exploratory use as a bridge or adjuvant while genetic or pharmacological interventions take effect. Beyond the strategies discussed in this review, cutting-edge technologies—particularly genome editing—are opening new avenues for tackling diseases driven by p63 mutations. However, in vivo validation and clinical trials will be essential before these innovations can be safely and effectively translated into patient care.

## Conclusion and future perspectives

In summary, p63 is indispensable for skin homeostasis and development, with its TAp63 and ΔNp63 isoforms playing distinct roles in regulating cell proliferation, differentiation, and stress responses. TAp63 functions as a tumor suppressor by inducing apoptosis and maintaining genomic stability, while ΔNp63 is crucial for preserving stem cell properties and regulating epithelial regeneration. This review has systematically elucidated p63’s multifaceted roles in skin biology, highlighting its involvement in cutaneous development, regeneration, differentiation, and its association with various p63 mutation-related skin disorders.

Looking forward, p63 research holds tremendous promise for clinical application while facing several challenges. A deeper understanding of isoform-specific, context-dependent p63 functions across different skin cell populations and developmental disease stages is needed. Investigating cross-talk between p63 and other key pathways (Wnt, Notch, BMP) will help unravel the complex regulatory networks governing skin homeostasis. Furthermore, elucidating molecular mechanisms underlying mutations in p63 and their associations with disease will be crucial for identifying precise therapeutic targets.

Current treatments for skin disease associated with mutations in p63 remain limited. Overcoming these limitations will require further knowledge of the nature of the disruptions in the molecular mechanisms associated with p63 mutations and the factors behind the heterogeneity of the patient phenotype. Although there has been considerable progress in recent years in the understanding of these molecular mechanisms, with novel perspectives provided by multidisciplinary integration, more profound research can still be carried out, and stem cell therapy represents a highly promising and potential research direction. Most importantly, advancing clinical translation represents a vital pathway for improving p63-associated skin disease treatment outcomes. Future work should include more clinical trials validating potential therapeutic targets’ efficacy and safety.

## Data Availability

The data that support the findings of this study are available from the corresponding author upon reasonable request.
